# Malposition of Central Venous Catheter Inserted under Ultrasound Guidance in Intensive Care Unit: A Case Series

**DOI:** 10.31729/jnma.4655

**Published:** 2020-07-31

**Authors:** Niraj Kumar Keyal, Sumal Thapa, Pooja Adhikari, Sanjeeb Kumar Yadav

**Affiliations:** 1 Department of Critical Care Medicine, B and C Medical College and Teaching Hospital, Birtamode, Nepal; 2 Department of Anaesthesia and Critical Care, B & C Medical College Teaching Hospital, Birtamode, Nepal

**Keywords:** *central venous catheter*, *internal jugular*, *malposition*, *subclavian*

## Abstract

Malposition of central venous catheter tip inserted into the subclavian and internal jugular vein is a rare unavoidable complication that can be decreased if inserted under ultrasound guidance. We report case series of three patients, two of subclavian and another of internal jugular inserted central venous catheter, in which the catheter malpositioned into ipsilateral internal jugular and subclavian vein respectively but had no effect on patient management. From this, we want to emphasize that the effect of malposition of central venous catheter tip depends upon the indication for which central venous catheter was inserted; it can be detected bedside by ultrasound and flush test.

## INTRODUCTION

Central venous catheter (CVC) is inserted in 8%^1^ of patients admitted to the hospital; it is technically challenging with known risk and complication. Complication like malposition, arrhythmia, infection, artery perforation, pneumothorax, hemothorax thrombosis has decreased by use of ultrasound during insertion of CVC.^2^ Malposition is defined as central venous catheter tip placement in a vein other than superior vena cava (SVC), or the right atrium, impingement with the lateral wall of SVC and arterial cannulation^3^ and occurs in 1.5 to 9.1% patients.^3,4^ We report a case series of three patients that had malposition of CVC inserted under ultrasound guidance that was detected by chest X-ray.

## CASE REPORT CASE 1

A 48-year old male with a past history of bronchial asthma, presented at the emergency department with the chief complaint of shortness of breath, fever, and productive cough for five days. A presentation in the emergency department, Glasgow coma scale (GCS) was 13/15, pulse rate 120 beats/per min, blood pressure (BP) 70/50 mmHg, respiratory rate 34 breaths/min, oxygen saturation 92% on 10 liters of oxygen. Chest examination showed bilateral crepts and wheeze. Cardiovascular and abdominal examination was normal. Arterial blood gas (ABG) analysis showed respiratory and lactic acidosis. He was diagnosed as an acute exacerbation of bronchial asthma, bilateral community-acquired pneumonia, septic shock with multiorgan dysfunction syndrome. The patient was intubated and resuscitated with balanced salt solution, noradrenaline, piperacillin-tazobactam, doxycycline.

**Figure 1.**
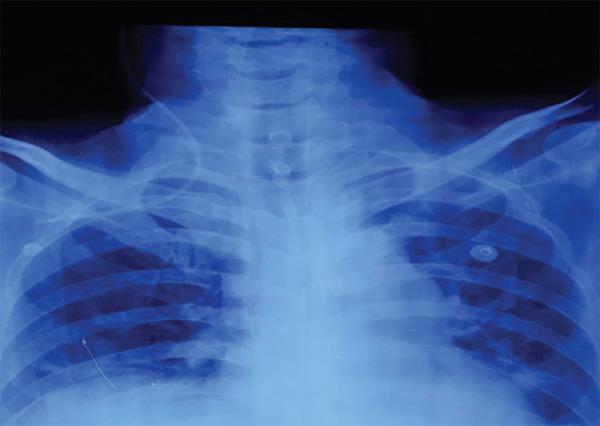
Malposition of right internal jugular catheter tip in the ipsilateral right subclavian vein.

Central venous catheter was inserted at right internal jugular vein (IJV) using the Selinger technique under ultrasound guidance in the first attempt; on the location of right IJV and free aspiration of blood, the guidewire was threaded freely through the needle without resistance and a 7French triple lumen catheter (Centrofix Trio V720, B Braun, Germany) was passed over the guidewire without any resistance but there was some resistance during withdraw of the guidewire. The catheter was fixed at 12 cm after free aspiration of blood from three ports of the catheter and checking the forward and backward flow, but invasive monitoring was not done. A chest X-ray showed a catheter tip at the ipsilateral right subclavian vein (Figure 1).

There was no problem with the transfusion of drugs. The patient was extubated on the fifth day and CVC was removed on the sixth hospital day. The patient was discharged on the tenth hospital day and was followed at the outpatient department after one week and had no new complaint.

## CASE 2

A 63-year old male with a past history of hypertension and diabetes mellitus under irregular medication presented with loss of consciousness, vomiting, headache. At a presentation in the emergency department, Glasgow coma scale (GCS) was 6/15, pulse rate 52 beats/per min, blood pressure (BP) 210/110 mmHg, respiratory rate 8 breaths/min, oxygen saturation 92% on 10 liters of oxygen. Chest, abdominal, and cardiovascular examination was normal. Arterial blood gas (ABG) analysis showed respiratory acidosis. He was diagnosed to have a left temporoparietal intracranial bleed with a mass effect on computed tomography of head. He was planned for emergency craniotomy and evacuation of hematoma. His routine investigations were found to be within normal limits.

**Figure 2.**
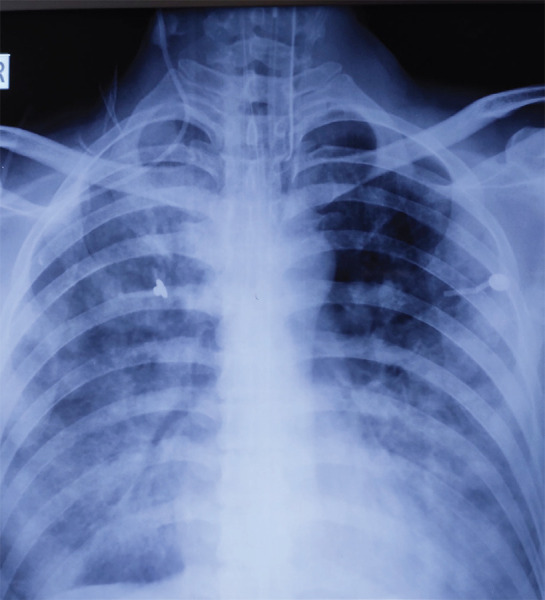
Malposition of the right subclavian vein catheter tip in the ipsilateral right internal jugular vein.

Central venous catheter was inserted at right subclavian vein (SCV) using the Seldinger technique with the supraclavicular approach under ultrasound guidance in the first attempt; on the location of right SCV and free aspiration of blood, the guidewire was threaded freely through the needle without resistance and a 7French triple lumen catheter (Centrofix Trio V720, B Braun, Germany) was passed over the guidewire without any resistance but there was some resistance during withdraw of the guidewire. The catheter was fixed at 12 cm mark after free aspiration of blood from three ports of the catheter and checking the forward and backward flow, but invasive monitoring was not done. A chest X-ray showed a catheter tip at ipsilateral IJV (Figure 2).

There was no problem with the transfusion of drugs. The patient was extubated on the third day and CVC was removed on the sixth hospital day. The patient was discharged on the tenth hospital day and was followed at the outpatient department after one week with the power of 3/5, 5/5 on right, and left upper and lower limb respectively.

## CASE 3

A 56-year old male with a past history of hypertension under irregular medication presented with loss of consciousness, vomiting, headache. At the presentation in the emergency department, the Glasgow coma scale (GCS) was 7/15, pulse rate 58 beats/per min, blood pressure (BP) 180/120 mmHg, respiratory rate 10 breaths/min, oxygen saturation 92% on 5 liters of oxygen. Chest, abdominal, and cardiovascular examination was normal. Arterial blood gas (ABG) analysis showed respiratory acidosis. Computed tomography of head showed subarachnoid hemorrhage. He was diagnosed to have a right-sided subarachnoid hemorrhage secondary to rupture of anterior communicating artery on computed tomography angiogram of the head. He was planned for emergency craniotomy and clipping if aneurysm. His routine investigations were found to be within normal limits.

**Figure 3.**
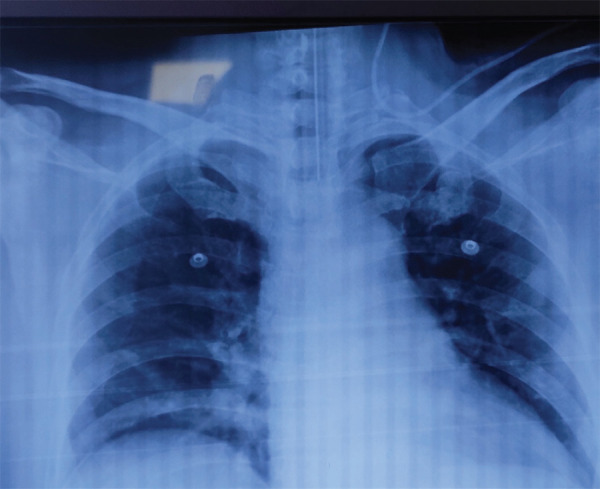
Malposition of left subclavian vein catheter tip in the ipsilateral left internal jugular vein.

Central venous catheter was inserted at left subclavian vein (SCV) using the seldinger technique with the supraclavicular approach under ultrasound guidance in the first attempt; on the location of left SCV and free aspiration of blood, the guidewire was threaded freely through the needle without resistance and a 7French triple lumen catheter (Centrofix Trio V720, B Braun, Germany) was passed over the guidewire without any resistance but there was some resistance during withdraw of the guidewire. The catheter was fixed at 12 cm mark after free aspiration of blood from three ports of the catheter and checking the forward and backward flow, but invasive monitoring was not done. A chest X-ray showed a catheter tip at ipsilateral IJV (Figure 3).

There was no problem with the transfusion of drugs. The patient was extubated on the second day and CVC was removed on the tenth hospital day. The patient was discharged on the fifteenth hospital day and was followed at the outpatient department after one week.

## DISCUSSION

Intravascular malposition of CVC is common on the left side than the right side and include IJV to ipsilateral SCV and vice versa, one SCV to contralateral SCV and vice versa, a reversal of the direction, internal mammary vein, azygos vein, hemizygous vein, superior intercostals, and thymic vein, coiling, twisting and extravascular malposition include pleural space, pericardium, mediastinum and catheter migration.^6^ Our patient also had malposition of SCV to ipsilateral IJV and IJV to SCV.

The possible reason for malpositions could be the change in the direction of the J tip of the guide wire during the procedure, excessive force applied during threading of guidewire or catheter, excessive length of guidewire insertion, the position of head and shoulder^2^ and in our patient increase angle between SCV and IJV may be the cause of malposition.

The effect of malposition depends to some extent on the indication of CVC. Manipulation of CVC was not done as the use of CVC was the administration of drugs and resuscitation in our patient; hemodynamic monitoring through CVC was not done as it is a dynamic parameter and less reliable.^6^ Guideline and studies^7^ have shown that malposition is decreased by use of ultrasound. But malposition occurred in our patient with the use of ultrasound by an experienced doctor.

Resistance during central venous catheter insertion, poor blood aspiration after insertion, excessively high CVP, and abnormal waveform may be indicative of catheter malpositioning^1,8^ but CVP monitoring was not done which may have missed early detection of malposition.

The possible ways to decrease malposition is to keep the guidewire J-tip directed caudad,^3^ insertion under fluoroscopic guidance, application of external pressure of IJV during threading of guidewire from SCV.^2^

The possible ways to detect malposition at the bedside are flush test, bedside ultrasound with agitated or non-agitated normal saline,^9^ electrographic guided tip placement, echocardiography IJV occlusion test, and chest x-ray but chest X-ray was only done in our patient.

Malposition of the central venous catheter is a preventable unavoidable complication that can be detected early by flush test and bedside ultrasound.

## Consent:

**JNMA Case Report Consent Form** was signedby the patient and the original article is attached withthe patient's chart.

## Conflict of Interest

**None.**
